# The complete chloroplast genome sequences of three Broussonetia species and comparative analysis within the Moraceae

**DOI:** 10.7717/peerj.14293

**Published:** 2022-10-31

**Authors:** Jinhong Yang, Qu Chu, Gang Meng, Weiqing Kong

**Affiliations:** Shaanxi Key Laboratory of Sericulture, Ankang University, Ankang, China

**Keywords:** *Broussonetia*, Moraceae, Chloroplast genome, Phylogeny, Comparative analysis

## Abstract

**Background:**

Species of* Broussonetia* (family Moraceae) are commonly used to make textiles and high-grade paper. The distribution of *Broussonetia papyrifera* L. is considered to be related to the spread and location of humans. The complete chloroplast (cp) genomes of *B. papyrifera*, *Broussonetia kazinoki* Sieb., and *Broussonetia kaempferi* Sieb. were analyzed to better understand the status and evolutionary biology of the genus *Broussonetia*.

**Methods:**

The cp genomes were assembled and characterized using SOAPdenovo2 and DOGMA. Phylogenetic and molecular dating analysis were performed using the concatenated nucleotide sequences of 35 species in the Moraceae family and were based on 66 protein-coding genes (PCGs). An analysis of the sequence divergence (pi) of each PCG among the 35 cp genomes was conducted using DnaSP v6. Codon usage indices were calculated using the CodonW program.

**Results:**

All three cp genomes had the typical land plant quadripartite structure, ranging in size from 160,239 bp to 160,841 bp. The *ribosomal protein L22* gene (*RPL22*) was either incomplete or missing in all three *Broussonetia* species. Phylogenetic analysis revealed two clades. Clade 1 included *Morus* and *Artocarpus*, whereas clade 2 included the other seven genera. *Malaisia scandens* Lour. was clustered within the genus *Broussonetia*. The differentiation of *Broussonetia* was estimated to have taken place 26 million years ago. The PCGs’ pi values ranged from 0.0005 to 0.0419, indicating small differences within the Moraceae family. The distribution of most of the genes in the effective number of codons plot (ENc-plot) fell on or near the trend line; the slopes of the trend line of neutrality plots were within the range of 0.0363–0.171. These results will facilitate the identification, taxonomy, and utilization of the *Broussonetia* species and further the evolutionary studies of the Moraceae family.

## Introduction

*Broussonetia* (family Moraceae) species are trees or shrubs producing economically valuable wood. They are typically native to eastern Asia but have been introduced to all of the Pacific Islands (Seelenfreund et al., 2011). Currently, there are five recognized species in this genus (Wang, Huang & Qin, 2012; Chung et al., 2017). Of these five, *Broussonetia papyrifera* L. is a pioneer species with strong stress tolerance. It usually grows on arid hillsides, in valleys, and on roadsides. The bark of *B. papyrifera* can be used to make textiles and high-grade paper, hence its common name “paper mulberry”. In 2019, the complete nuclear genome of *B. papyrifera* was sequenced and the genetic basis for its adaptability in paper-making, animal feed, and medicine was revealed (Peng et al., 2019). *Broussonetia kazinoki* Sieb. and *Broussonetia kaempferi* Sieb. are both shrubs. Together with *B. papyrifera*, all three species belong to the tribe Broussonetieae Gaud (Zhang & Cao, 1998). Their leaf, bark, fruit, and latex can be used as raw materials for medicines (Cao et al., 2020).

An artificial, interspecific hybrid (hybrid paper mulberry) was crossbred between *B. kazinoki* and *B. papyrifera*. The leaves of the resulting plant are widely used as fodder because they are rich in protein (Si et al., 2018). The hybridization of *Broussonetia* may occur rarely in nature. *B.* × *kazinoki* is a natural hybridization between *Broussonetia monoica* Hance (synonym: *B. kazinoki*) and *B. papyrifera*, is known as “kōzo” in Japan and “daknamu” in Korea, and it is used as the main material for making traditional paper (Kuo et al., 2022).

The distribution of *B. papyrifera* is thought to be related to human migration throughout history (Payacan et al., 2017). A tree native to South China and Southeast Asia, *B. papyrifera,* was brought to Polynesia by Austronesian-speaking travelers as a source of bark fiber. It is now distributed as far as Easter Island and is now homogeneous in the Pacific, representing a rich germplasm resource (Chang et al., 2015). *B. papyrifera* also shows dispersal patterns across the vast Pacific region on the basis of ribosomal RNA sequences and inter-simple sequence repeat (ISSR) markers. These findings are in agreement with current archaeological evidence (Matisoo-Smith, 2015).

The chloroplast (cp) is an important organelle found in green plant cells with approximately 10,000 DNA copies per leaf cell (Bendich, 1987). The sequence of the cp genome is an appropriate molecular marker for plants, being restricted to both lower and higher plants. Recently, genes or inter-gene spacers in the cp genome have been used in plant molecular systematic studies (Wu et al., 2018; Li et al., 2020; Li et al., 2021). Next-generation sequencing (NGS) may significantly reduce the time and cost of obtaining abundant nucleotide sequences. Approximately five percent of the reads obtained by NGS of plant species are derived from the chloroplast, which is perfectly amenable to cp genome assembly (Bakker et al., 2016). To-date, a total of 117 cp genomes from 71 species of Moraceae have been sequenced.

The cp genomes of six species of the *Broussonetia* alliance were assembled and used to identify the origin of *B.* ×* kazinoki* and to re-examine the taxonomic proposition of the *Broussonetia* alliance (Kuo et al., 2022). The current research on the cp genome of the hybrid paper mulberry supports a close genetic relationship with *B. kazinoki* and the maternal genetics of the cp genome (Zhang et al., 2022). Our research reports on the sequencing information and characterizations of the complete cp genomes of *B. papyrifera*, *B. kazinoki,* and *B. kaempferi*, which are all *Broussonetia* species found on the Chinese mainland (Zheng et al., 2002). We also analyzed the phylogenetic relationships, differentiation times, codon usage pattern, and natural selection pressures within the Moraceae family. This information may be helpful to better understand the evolutionary history and driving forces of the Moraceae.

## Materials & Methods

### Sample collection, DNA extraction, and genome sequencing

**Figure 1:**
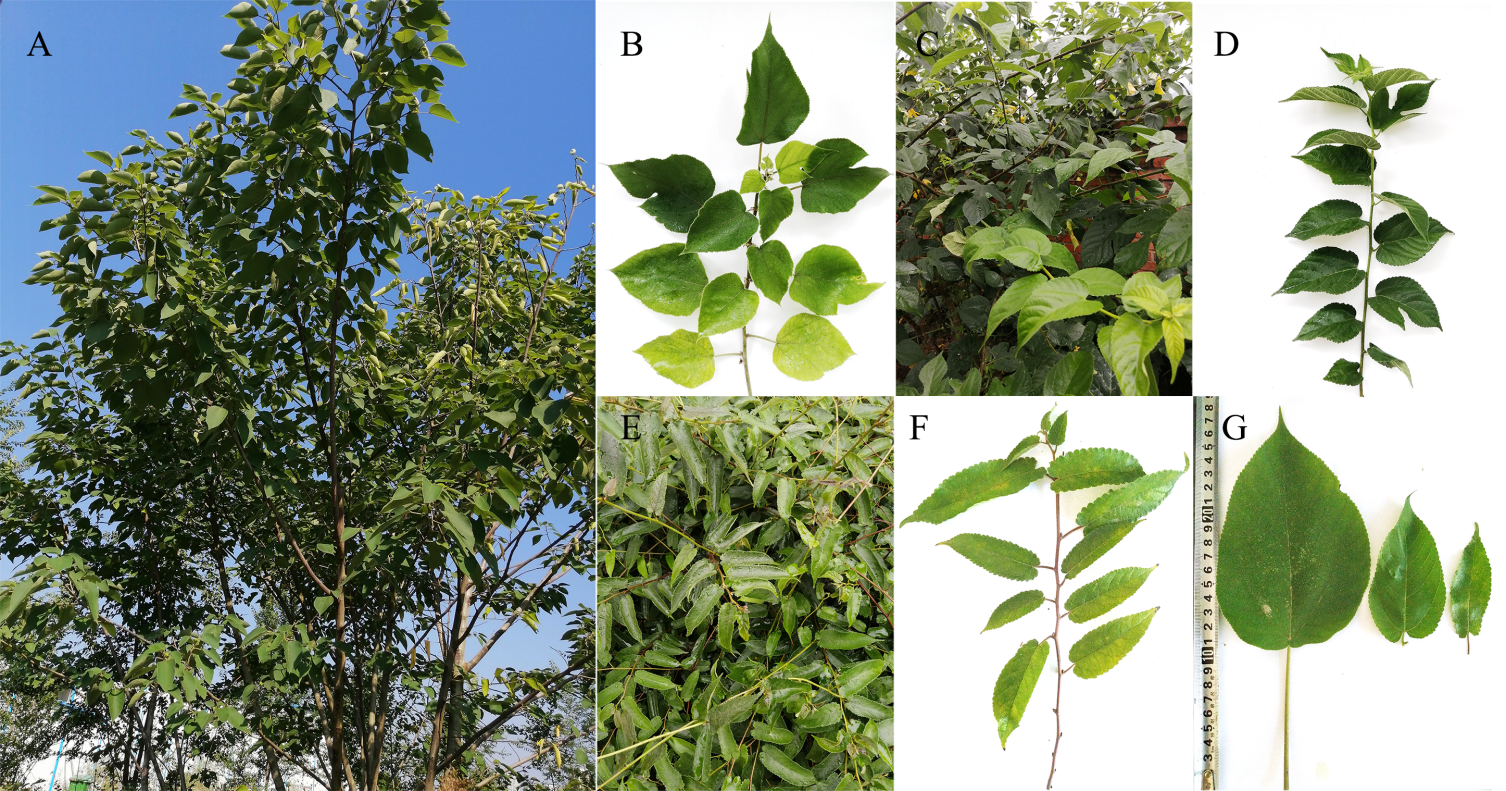
Morphology of three *Broussonetia* species. (A, B) Plant and branch of *B. papyrifera*. (C, D) Plant and branch of *B. Kazinoki*. (E, F) Plant and branch of *B. kaempferi*. (G) Leaf morphology of three *Broussonetia* species.

Fresh leaves of *B. papyrifera* and *B. kazinoki* were sampled from a single plant of each species growing in Ankang (108°58′55′E, 32°41′50′N), Shaanxi Province, China, on September, 2015. The fresh leaves of *B. kaempferi* were sampled from a single tree growing in Liuzhou (109°26′59′E, 24°17′12′N), Guangxi Province, China, on April, 2017. The voucher specimens of the three species were planted in the Sericultural Research Base of Ankang University (Fig. 1).

A modified high-salt method was used to extract the genomic DNA from the leaves (Shi et al., 2012). The resulting genomic DNA was then fragmented and indexed by barcoding. The paired-end libraries, with an insert size of ∼350 bp, were then constructed in accordance with the standard Illumina protocol and the sequencing was carried out on the HiSeq 2000 platform with 125-bp paired-end reads (Illumina Inc., San Diego, CA, USA).

### Genome assembly and annotation

The chloroplast reads were isolated from the raw reads using the bowtie2 software (Langmead & Salzberg, 2012) with a very sensitive local model and cp genome of *Ficus racemosa* L. (Mao & Bi, 2016) used as a reference. The resulting reads were assembled using SOAPdenovo2 with Kmer = 63 (Luo et al., 2012). Then, all of the contigs were mapped to the reference cp genome using BLAT (Kent, 2002) to identify their position and direction. Sequences with ambiguous alignment were trimmed manually and were regarded as gaps. The gaps were filled by the consensus sequence, which was generated with the model implemented in MAQ software (Li, Ruan & Durbin, 2008). The process was repeated until the reference genome was fully supported. SAMtools (Li et al., 2009) was used to parse the depth of assembly sequences.

The preliminary annotations of *B. papyrifera*, *B. kazinoki*, and *B. kaempferi* cp genomes were performed using the online automatic annotator, DOGMA (Wyman, Jansen & Boore, 2004). The protein coding genes (PCGs) and rRNA genes were then verified using BLASTN searches (e-value cutoff = 1e−10) against other Moraceae family cp genomes to ensure accurate annotations (Chen et al., 2015). The start and stop codons, or intron and exon junctions, of each annotated PCG were manually compared with the cp genomes of *F. racemosa* and *Morus mongolica* Schn. using the check_annotations.py module (Jin et al., 2020). The tRNA genes were confirmed using tRNAscan-SE 1.21, which specified that mitochondrial/cp DNA was the source (Schattner, Brooks & Lowe, 2005).

### The analysis of *RPL22* deficiency or transfer in genus *Broussonetia*

The primers, F22 (5′-GCAAACCAAAGAGAATGATGAC-3′), and R22 (5′-CGAGCGTCTACCATTATACCTAC-3′), were designed for the amplification of the *RPS3*–*RPS19* region, including the inter-genic (IG) regions of *RPS3*–*RPL22* and *RPL22*–*RPL19*, and the *RPL22* gene. They were used to identify *RPL22* gene deficiency in the cp genome. The PCR amplifications of the *RPS3*–*RPS19* region were carried out with the genomic DNA of *B. papyrifera*, *B. kaempferi*, and *B. kazinoki*, as templates. The *RPS3–RPS19* region of hybrid *B. kazinoki* ×* B. papyrifera*, and *M. mongolica* were amplified at the same time. The PCR products were then cloned and sequenced. The resulting sequences were then aligned using ClustalX1.83 software (Thompson et al., 1997), and the corresponding results were adjusted manually according to the *RPS3*, *RPL22*, and *RPS19* gene borders.

The primers, rpl22f (5′-ATAACCCCGTCCTCGAGCTT-3′) and rpl22r (5′-AGAAGAGAAGGACCAAGCGA-3′), were designed inside the *RPL22* gene, according to *F. racemosa* and *M. mongolica*, and were used to identify whether the *RPL22* gene was transferred to the nuclear genome. The *RPL22* genes of *M. mongolica* were amplified simultaneously as a control. The amino acid sequence of *RPL22* from *F. racemosa* and *M. mongolica* were subjected to TBLASTN analysis of the whole-genome sequence of *B. papyrifera* and the raw data (Gertz et al., 2006).

### Phylogenetic analysis

To illustrate the phylogenetic relationships among members of the family Moraceae, 32 complete cp genomes were downloaded from GenBank (Table S1). Three species *Rosa chinensis* Jacq., *Rosa minutifolia* Engelm. and *Rosa rugosa* Thunb. from the family Rosaceae were used as the outgroups. The sequences of 66 PCGs present in all the 38 species (35 plus three outgroup species) were extracted using Python 3.6. Then, each of the 66 PCGs were aligned, accounting for frame shifts and stop codons, by MACSE with default settings (Ranwez et al., 2018). Phylogenetic analyses were performed using the concatenated nucleotide sequences by both the Maximum-Likelihood (ML) and Bayesian Inference (BI) methods. The generalized time reversible (GTR) with invariable sites (+I) discrete gamma (+G) model was selected by model test applying the Akaike information criterion (AIC) and Bayesian information criterion (BIC) using jModelTest (Darriba et al., 2012). ML phylogenies were inferred using RAxML 8.2.12 software, the bootstrap analysis was performed with 1,000 replications (Stamatakis, 2014). BI phylogenies were inferred using MrBayes 3.2.7 (Ronquist et al., 2012), with Markov chain Monte Carlo (MCMC) algorithm of 1,000,000 generations, sampled every 1,000 generations until convergence. The first 25% of the trees were discarded as burn-in, while the remaining trees were used to generate the consensus tree.

### Divergence time estimation

The RAxML tree and software MCMCTREE of PAML (Phylogenetic Analysis of Maximum Likelihood) (version 4.9j) was used for divergence time analysis (Yang, 2007). We estimated the divergence time under the relaxed clock and the Hasegawa-Kishino-Yano 1985 (HKY85) nucleotide substitution model. The nucleotide substitution rate was set as (*r* = 1. 368 ×10^−9^, rgene gamma =2, 14.6, 1) gamma distribution (Muse, 2000; Xu et al., 2012). The two primary calibration points in our analyses were: (1) the divergence between the Moraceae and the Rosaceae, 42–161 million years ago (Mya), according to http://www.timetree.org; and (2) the divergence between *Morus alba* L. and *Morus notabilis* Schn*.*, 6–17 Mya, based on genome sequence estimates (Jiao et al., 2020).

The MCMC method was run for 2,000 generations as burn-in, then sampled every ten generations until a total of 20,000 samples had been generated. Convergence at each node was determined using Tracer v1.7 by confirming the effective sample sizes (ESS) above 200, with the 95% highest probability density (HPD) accepted (Rambaut et al., 2018).

### Natural selection event analysis

The natural selection events can be measured by non-synonymous substitutions (dN) and synonymous substitutions (dS). The observation dN >dS suggest positive selection, otherwise it will be negative selection. The value of dN/dS was calculated by two methods: (1) using HyPhy 2.2.4 software, the unrooted phylogenetic tree, the Branch-site model, and the Muse-Gaut 1994 (MG94) codon substitution model (Pond, Frost & Muse, 2005); and (2) using program CodeML in PAML and the pairwise comparison matrix of 35 concatenated nucleotide sequences (Yang, 2007).

### PCG divergence and indices of codon usage

The analysis for the sequence divergence (pi) of each PCG among the 35 cp genomes was conducted using DnaSP v6 software (Rozas et al., 2017). The amino acid composition and relative synonymous codon usage (RSCU) values were calculated using Mega 11 (Tamura, Stecher & Kumar, 2021). The effective number of codons (ENc) is widely used as a measure of codon usage bias (CUB). GC3S indicated the GC content at the third synonymously-variable coding position and excluded Met, Trp, and the three stop codons, which are indicators of the level of nucleotide composition bias (Wright, 1990; Ahmad et al., 2013). ENc-plot (ENc *vs* GC3S) is a useful indicator of the factors affecting codon usage (Comeron & Aguadé, 1998; Peden, 1999). The values of ENc and GC3S were calculated using the CodonW program in the Mobyle server (https://mobyle.rpbs.univ-paris-diderot.fr/cgi-bin/portal.py#welcome). The values of GC12 (the GC contents at the first and second positions) and GC3 (GC contents at the third position) were also obtained by this method.

## Results

### Genome assembly and features

The mean depth of the final assembled *B. papyrifera*, *B. kazinoki*, and *B. kaempferi* cp genomes were approximately 484.2-fold, 295.1-fold, and 608.2-fold, respectively (Table S2). Their lengths were 160,239 bp, 160,841 bp, and 160,592 bp, respectively (Table 1). The lengths were all shorter than those of *Broussonetia kurzii* Hook and *Broussonetia luzonica* Blanco, but longer than those of the genus *Morus*. All three cp genomes had the quadripartite structures typical of land plants, including a large single-copy (LSC) region, a small single-copy (SSC) region, and two inverted-repeat (IR) regions.

**Table 1:** The chloroplast genome features of five *Broussonetia* species.

Attribute	*B. papyrifera*	*B. kazinoki*	*B. kaempferi*	*B.luzonica*	*B. kurzii*
Genome size/GC content	160,239/35.83	160,841/35.73	160,592/35.64	162594/35.66	162170/35.66
LSC size/ GC content	88,622/33.5	89,066/33.34	89,001/33.22	89980/33.29	90174/33.24
SSC size/ GC content	19,919/28.5	20,093/28.47	19,997/28.3	19560/28.52	20150/28.51
IR size/ GC content	25,849/42.66	25,841/42.68	25,797/42.67	2652742.32	25923/42.66

The GC contents of *B. papyrifera*, *B. kazinoki*, and *B. kaempferi* were 35.83%, 35.73%, and 35.64%, respectively. As with other members of the Moraceae, the GC content distribution of the three *Broussonetia* cp genomes was also uneven, being highest in the IR region, intermediate in the LSC region, and lowest in the SSC region (Table 1).

**Figure 2:**
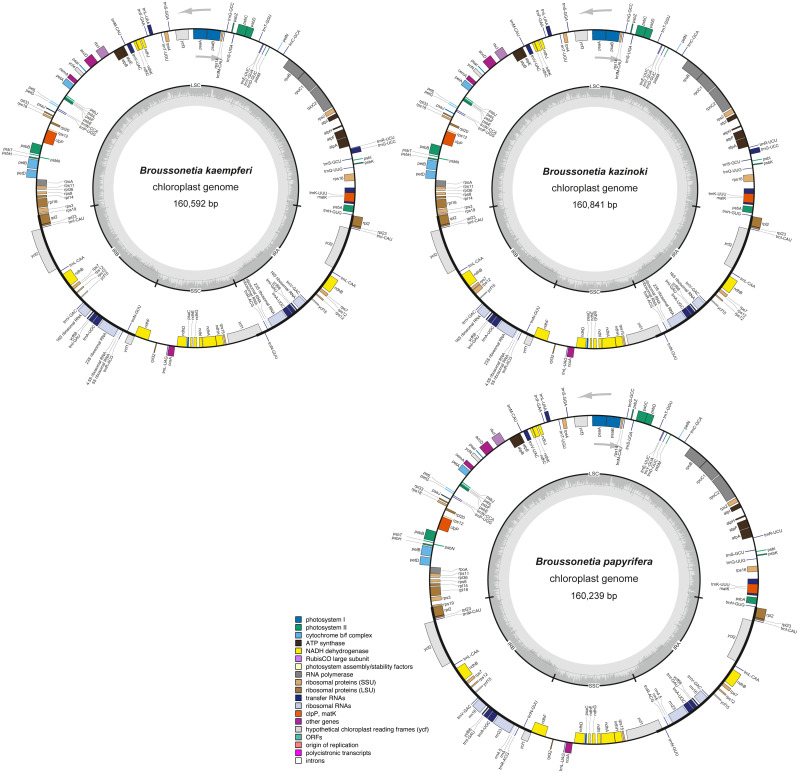
The cp genome structure of three *Broussonetia* species. The colored bars indicate different functional groups. The dark gray inner circle corresponds to GC content, the light-gray to AT content. All three genomes were composed by the large single copy regions (LSC), small single copy regions (SSC) and the inverted repeat regions of IRA and IRB. Genes drawn inside and outside the circle are transcribed clockwise and counter-clockwise, respectively.

Overall, 131 genes were found in each of the three *Broussonetia* cp genomes, namely 86 PCGs, 36 tRNAs, and eight rRNAs (Table S3), arranged in the same linear order as in other species of the Moraceae, except for gene *RPL22*, which was absent from the three *Broussonetia* cp genomes (Fig. 2). Of all these genes, seven PCGs, seven tRNAs, and four rRNAs were duplicated in the IR regions. Additionally, ten PCGs (*atpF*, *ndhA*, *ndhB*, *petB*, *petD*, *RPL2*, *RPL16*, *rpoC1*, *RPS16*, and *ycf68*) and six tRNAs (*trnI-GAU*, *trnG-UCC*, *trnK-UUU*, *trnL-UAA*, *trnV-UAC,* and *trnA-UGC*) contained one intron, whereas three PCGs (*RPS12*, *ycf3*, and *clpP*) had two introns (Table S3).

### The absence of the *RPL22* gene from the genus *Broussonetia*

**Figure 3:**
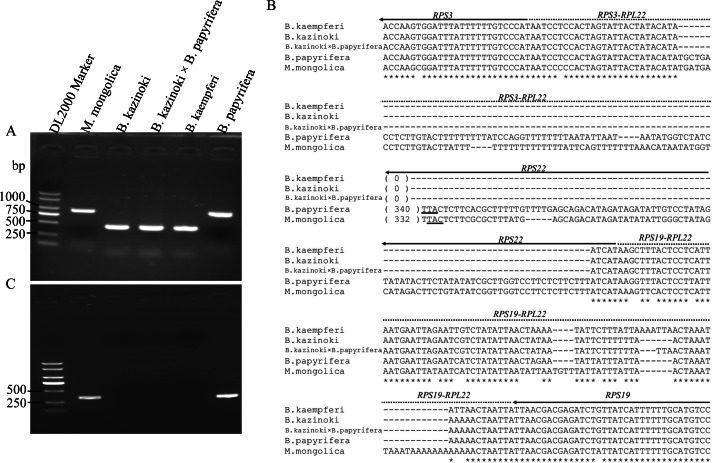
The detection and analysis of *Broussonetia RPL22* gene. (A) PCR amplification of the RPS3–RPS19 region. (B) The alignment analysis of RPS3–RPS19 region, the arrow indicated the direction of gene transcribed, the underlined TTA indicated the stop codon. (C) PCR amplification of the RPL22 region.

Agarose gel electrophoresis showed that the PCR amplification products of the F22 and R22 primers were different lengths (Fig. 3A). The sequences of the clones were the same as those in the HiSeq 2000 platform, which confirmed that the *RPL22* gene is a truncated pseudogene in *B. papyrifera* and was lost in *B.kaempferi*, *B.kazinoki*, and the hybrid *B. kazinoki* × *B. papyrifera* chloroplast genome (Fig. 3B).

The PCR amplification products of the rpl22f and rpl22r primers contained no expected products, indicating that there was no transfer of *RPL22* in *B. papyrifera*, *B. kaempferi*, *B. kazinoki*, and hybrid *B. kazinoki* × *B. papyrifera* (Fig. 3C). The results of TBLASTN analysis showed that no *RPL22* gene was detected in the *B. papyrifera* genome, too. Also, further gene content analysis showed that *RPL22* gene is lacked in other two *Broussonetia* plants (*B. luzonica* and *B. kurzii*), and *Malaisia scandens* Lour., a plant of genus *Malaisia* family Moraceae.

### Phylogenetic and molecular dating analysis

**Figure 4:**
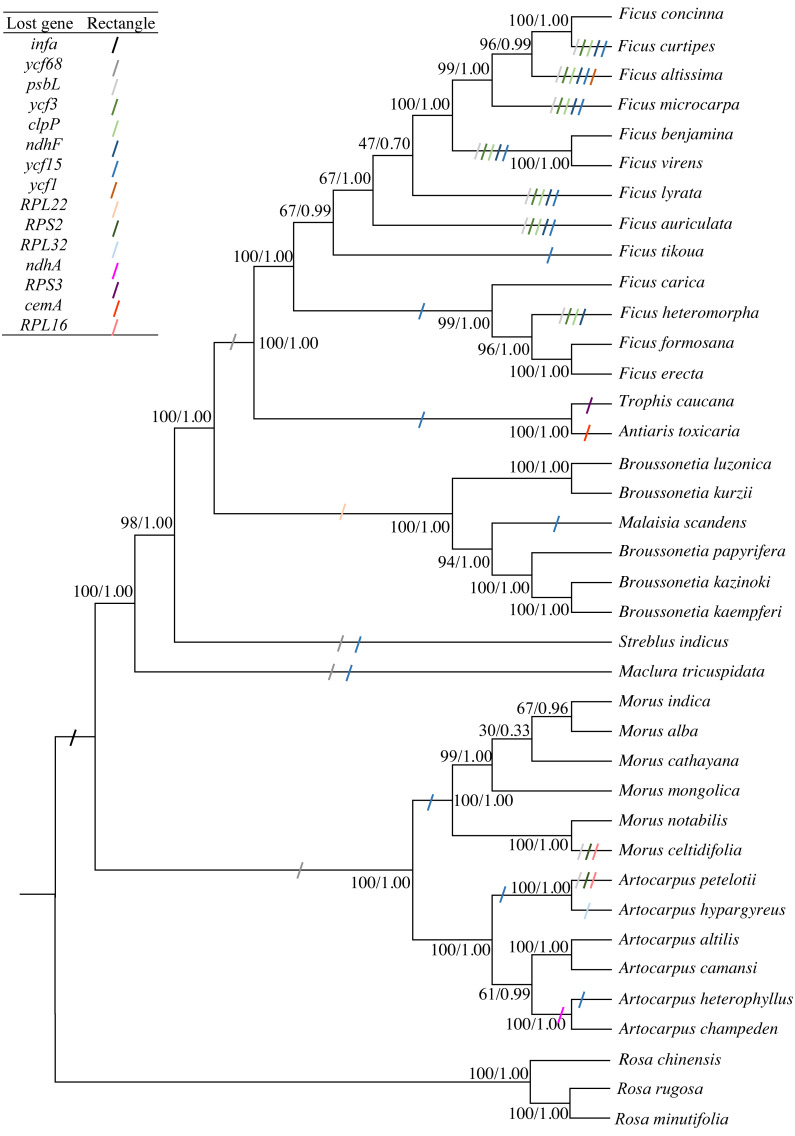
Phylogenetic trees constructed by maximum-likelihood (ML) and Bayesian inference (BI) methods. Three *Rosa* species were used as the outgroups. Numbers (before/after) near the nodes indicate ML bootstrap support and Bayesian posterior probability values. Colored rectangles indicate the putative gene losses.

The concatenated sequence, including 66 PCGs that were 56,217 bp in length and had 6,847 diverse loci, was used to construct the phylogenetic tree. Both ML and BI phylogenetic trees had two clades: clade 1 included *Morus* and *Artocarpus*, whereas clade 2 included the other seven genera. The three *Broussonetia* species in this study were clustered into one branch, and *M. scandens* was aggregated within this branch. *B. luzonica* and *B. kurzii* were present in the sister branch (Fig. 4). The genus *Broussonetia* was the earlier diverging lineage than the genera *Ficus*, *Trophis* and *Antiaris*, later than genus *Streblus*. Fifteen genes (*infA*, *psbL*, *ycf3*, *clpP*, *ndhF*, *ycf1*, *RPS2*, *RPL16*, *RPL32*, *RPS3*, *RPL22*, *ndhA*, *cemA*, *ycf68*, and *ycf15*) were lost to differing degrees from the 35 cp genomes (Fig. 4).

The likelihood of the best-scored ML tree was 137,376 and the bootstrap support values in all the nodes between the genera were greater than 90%, reaching 100% in five cases (Fig. 4). At the species level, the values at the partial nodes (six out of 35) were less than 90%, especially in the species of genus *Morus*. The values at the nodes of (*Morus cathayana* Hemsl. (*M. alba*, *Morus indica* L.)) were 30 and 67, respectively. The BI tree showed similar topologies with the ML tree. The three clades of *M. cathayana*, (*M. alba*, *Morus indica*) and *M. mongolica* in the BI tree could not be effectively distinguish (Fig. 4), which may be caused by the interspecific free hybridization within the genus *Morus*.

**Figure 5:**
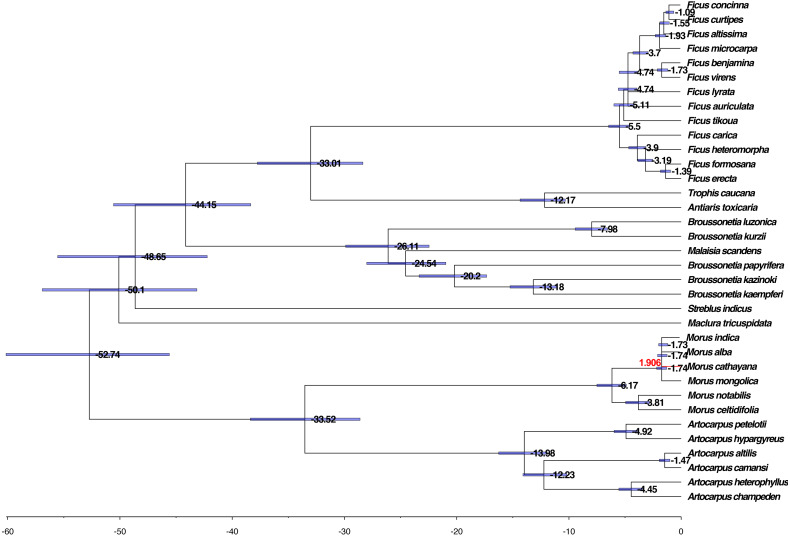
Phylogenomic analysis of the divergence time and natural selection. The branch lengths are proportional to the estimated times. Numbers denoted at each node are inferred divergence times (million years). Blue bars are 95% highest probability density (HPD) accepted. The red branch line indicates dN/dS >1.

We further evaluated the molecular clock based on the phylogenetic tree, HKY85 nucleotide substitution, and two calibration points, 42–161 Mya for the divergence between the Moraceae and the Rosaceae and 6–17 Mya for the divergence between *M. alba* and *M. notabilis*. The mean and 95% highest posterior density (HPD) divergence times were mapped onto the phylogenetic tree. The common ancestor of Moraceae occurred before 52.74 Mya, whereas divergence between genus took place within the range of 33.01–50.10 Mya among the genera. The divergence of *Broussonetia* took place around 26.11 Mya. This was much earlier than the divergence of *Morus*, *Artocarpus*, and *Ficus*, which diverged approximately 5.50–19.98 Mya (Fig. 5).

### Natural selection event analysis

The dN/dS ratios of all branches and species calculated using the unrooted tree were less than one except for *M. cathayana*, a wild mulberry species unique to China, which had a ratio of 1.906 (Fig. 5). The dN/dS ratios based on the pairwise comparison matrix of concatenated nucleotide sequences showed that the highest average value for any genus was 0.519 for *Morus*, compared with 0.229 for *Broussonetia*, 0.241 for *Ficus*, and 0.413 for *Artocarpus* (Tables S4 –S7). The ratio between *M. cathayana* and *M. mongolica* was 2.705 (Table S4), which was the highest ratio occurring in the genus *Morus*.

### PCG divergence and indices of codon usage

The analysis of sequence divergence among the PCGs of the 35 cp genomes showed that the pi values were in the range of 0.0005 (*psbF*)-−0.0419 (*RPS16*) with a mean of 0.0174, indicating small differences among the PCGs. The pi values of PCGs in the SSC region were all greater than 0.01, with a mean of 0.0226, which was much higher than those in the IR regions, all of which were below 0.01 (Fig. S1).

A total of 26,288–26,306 codons were identified in the cp genome of three *Broussonetia* species; these were used in RSCU analysis. The results showed that the most commonly used codons consisted of mostly A and U (*i.e.,* AUU for Ile, AAA for Lys, UUU for Phe, and AAU for Asn). The codons that were infrequently used consisted of more G and C (*i.e.,* GGC for Gly, CGC and CGG for Arg, CUG and CUC for Leu, GCG for Ala, and CCG for Pro). In addition, there were 30 codons with RSCU >1; among them, 29 codons ended with A or U and one codon (UUG for Leu, 1.20–1.21) ended with G (Table S8).

**Figure 6:**
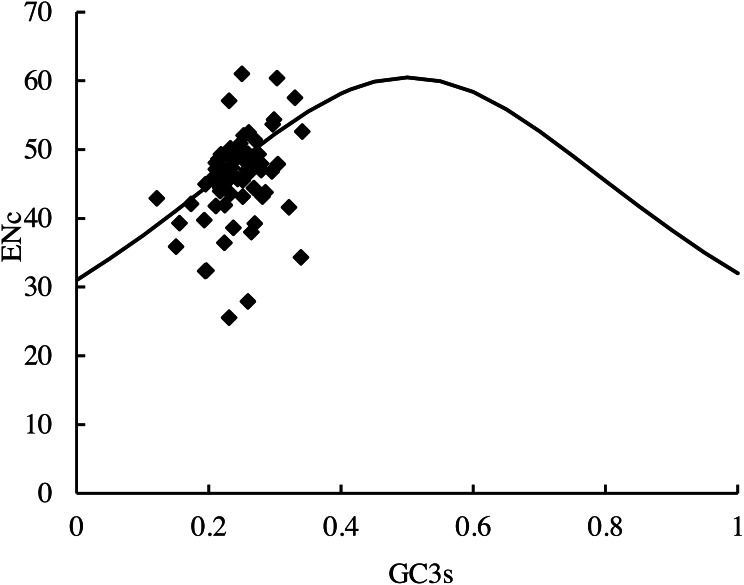
ENc-plots (ENc *vs* GC3S) of 66 protein-coding genes (PCGs). Solid lines are expected ENc from GC3S.

The distribution of the average ENc values of the PCGs in this study was 25.6 (*RPL36*)–61.0 (*psbF*). The distribution of the average GC3S value was 12.10%–34.14%. The distribution of the majority of the genes in the ENc-plots fell on or near the trend line, except for *psbF* (ENc =61), which was above the curve. In addition, some genes (*petN*, *psbI*, *psbJ*, *RPL33*, and *RPL36*) fell far below the expected line, suggesting that the codon usage bias could be affected by many factors (Fig. 6).

Further analysis of the base composition showed that the distribution of the average GC content for GC12 was 33.55%–56.08%, and for GC3 was 14.32%–36.73%. The distribution range for each was relatively narrow. The neutrality plots of GC12 *versus* GC3 for the 35 species showed that the distributions of the genes deviated from the diagonal line (slopes = 1) and the slopes of the trend lines ranged from 0.036 to 0.171. The correlation coefficient ranged from 0.032 to 0.151 (Table 2), which indicated that there were no correlations between GC12 and GC3 for the genes.

**Table 2:** The neutrality analysis of GC12 versus GC3 for the 35 species.

Species	Slope	Intercept	Relative index	Species	Slope	Intercept	Relative index
*Antiaris toxicaria*	0.108	0.399	0.101	*Ficus erecta*	0.070	0.408	0.063
*Artocarpus altilis*	0.131	0.391	0.107	*Ficus formosana*	0.073	0.408	0.065
*Artocarpus camansi*	0.138	0.389	0.113	*Ficus heteromorpha*	0.069	0.409	0.063
*Artocarpus champeden*	0.131	0.392	0.110	*Ficus lyrata*	0.048	0.415	0.043
*Artocarpus heterophyllus*	0.093	0.403	0.079	*Ficus microcarpa*	0.062	0.410	0.056
*Artocarpus hypargyreus*	0.060	0.413	0.052	*Ficus tikoua*	0.083	0.405	0.075
*Artocarpus petelotii*	0.036	0.420	0.032	*Ficus virens*	0.076	0.407	0.067
*Broussonetia kaempferi*	0.071	0.408	0.060	*Maclura tricuspidata*	0.171	0.382	0.151
*Broussonetia kazinoki*	0.048	0.415	0.041	*Malaisia scandens*	0.101	0.400	0.092
*Broussonetia kurzii*	0.047	0.415	0.045	*Morus alba*	0.104	0.400	0.092
*Broussonetia luzonica*	0.048	0.415	0.044	*Morus cathayana*	0.111	0.398	0.098
*Broussonetia papyrifera*	0.120	0.395	0.107	*Morus celtidifolia*	0.105	0.400	0.094
*Ficus altissima*	0.067	0.409	0.060	*Morus indica*	0.102	0.400	0.092
*Ficus auriculata*	0.070	0.408	0.063	*Morus mongolica*	0.106	0.400	0.094
*Ficus benjamina*	0.069	0.408	0.061	*Morus notabilis*	0.120	0.395	0.107
*Ficus carica*	0.089	0.403	0.080	*Streblus indicus*	0.123	0.394	0.101
*Ficus concinna*	0.083	0.405	0.074	*Trophis caucana*	0.145	0.389	0.138
*Ficus curtipes*	0.079	0.406	0.070				

## Discussion

We determined the complete cp genome of three *Broussonetia* species, and found the *RPL22* gene was incomplete in, or completely missing from, the cp genome of all *Broussonetia* species. These results are consistent with those from Kuo et al. (2022). Moreover, the *RPL22* gene was not transferred to the nuclear genome and may be used as a potential molecular marker to identity the *Broussonetia* alliance from the family Moracea. Some gene fragments were found to have been functionally transferred from chloroplasts to the nuclear genome, such as *RPL22* of the legume pea, which was found to have been transferred to its nuclear genome (Gantt et al., 1991). In addition, *nptII* (an exogenous resistance gene integrated into the tobacco plastid), *psbA* of rice (Oryza sativa L.), and *rpoA* of the moss *Physcomitrella patens* Hedw., were all found to have been transferred from their cp genome to their nuclear genome (Yu et al., 1997; Cove, 2000; Huang, Ayliffe & Timmis, 2003; Stegemann & Bock, 2006). These proteins encoded by the now-nuclear genes were then transported to the cp to perform their functions (James, 2003).

The gene absent events of all the cp genomes used in this study were analyzed. A total of 15 genes were lost from the 35 Moraceae cp genomes to different degrees (Fig. 4). These results are consistent with the cp genome undergoing some changes (gene loss, transfer, rearrangement) over a long-term evolutionary process (Huang, Ayliffe & Timmis, 2003; Wang et al., 2018). The *infA* gene was lost many times (Millen et al., 2001), and was deleted from most of the *Rosid* cp genome during angiosperm evolution (Kong & Yang, 2016; Tao et al., 2017). As a result, the *infA* gene was lost from all 35 studied species. The *psbL* gene was lost from 12 of the species studied, including ten from the genus *Ficus* and each one from *Morus* and *Artocarpus*. The deletion of the *ycf3*, *clpP,* and *ndhF* genes was observed in eight species of the genus *Ficus*. There were up to five missing genes in *Ficus altissima* Bl.; the *ycf1* gene was also considered. *Ycf1*, *ycf2*, *RPL22*, *RPL23,* and *accD* were also lost from some angiosperms (Jansen et al., 2007; Guisinger et al., 2010). The chloroplast genes could not be easily discarded in photosynthetic species and were functionally removed by some unknown mechanism. Therefore, further studies are needed to determine whether the absent genes may be transferred to the nuclear genome or functionally replaced by nuclear genes (Ueda et al., 2008).

The phylogenetic relationship among 35 Moraceae species was studied in this article. *M. scandens*, which had previously been identified as a species of *Trophis* or *Caturus* (Green, 1993), was clustered within the genus *Broussonetia* with a very high bootstrap value. This was consistent with earlier studies (Chung et al., 2017; Kuo et al., 2022). There may be two reasons for this situation. First, *M. scandens* should have been located within the genus *Broussonetia* and the deletion of the *RPL22* gene from both *Broussonetia* spp. and *M. scandens* supports this view. Second, is related to the sample collection region (Fernández-Mazuecos & Vargas, 2010; Hazzi et al., 2018). The leaf samples of the three *Broussonetia* species in this study and *M. scandens* were all from the Chinese mainland, but the other two *Broussonetia* species (*B. luzonica* and *B. kurzii*) were sampled from Philippines and Thailand (Kuo et al., 2022).

The divergence of *Broussonetia* occurred long before that of the other three genera, which indicated that *Broussonetia* may exhibit more evolutionary differences in response to geographical distribution. The most common variant of *B. papyrifera* found in the Pacific region has a clear Taiwanese origin, and Taiwan harbored 19 haplotypes (total 48), of which 16 were endemic (Matisoo-Smith, 2015). In addition, the haplotype distribution based on *ndhF-rpl32* sequence also showed high differentiation of *B. monoica* from different regions (Kuo et al., 2022). The results all provide strong evidence of the evolutionary history of the *Broussonetia* genus.

The dN/dS value can reflect natural selection events (Mugal, Wolf & Kai, 2014). The dN/dS ratios among *Morus* and *Artocarpus* are higher than for other Moraceae genera, which indicated that the clade of *Morus* and *Artocarpus* had undergone positive selection. This may have been caused by the artificial selection of mulberry trees for their use in raising silkworms. The results also showed that, at the whole-chloroplast protein level, purifying (negative) selection is the major strategy in use by the other genera (Hershberg & Petrov, 2008).

The CUB may be affected by many factors, such as DNA variation under natural selection, tRNA abundance, gene sequence length, GC content, and protein translation efficiency, etc (Qin et al., 2013). The study of CUB is not only helpful in explaining the evolutionary pattern among species, but also has great significance in terms of gene expression, vector construction, and the analysis of unknown functional genes. The RSCU value is frequently observed for a codon and divided by the expected frequency, which is an important index of CUB (Sharp & Li, 1986). The RSCU values in the three *Broussonetia* species exhibited similar preferences, which is consistent with previous studies, showing that closely related species generally have similar RSCU values (Athey et al., 2017; Zhang et al., 2017; Gao et al., 2018; Peng et al., 2020). Meanwhile, A/U ending codons were preferred with higher RSCU (>1) values, as observed in the cp genomes of other land plants (Asaf et al., 2018; Song et al., 2022). These results indicated that those codons are used more frequently than expected, which may be driven by the high A/U content of the cp genome (Zhou, Long & Li, 2008; Gao et al., 2018).

The Enc-plot is a useful tool for studying the factors affecting codon usage, such as mutation or natural selection (Liu, 2013). Most of the 66 genes from the cp genomes of the 35 species used in this article followed the trend line, which indicated that the characteristics of codon usage were random, showed a weak bias, and that the base composition bias on the 3rd codon was the main influencer of the CUB. These results are similar to the ENc plot of the Asteraceae family (Nie et al., 2014). Meanwhile, several genes fall far below the line in the plot, indicating that selection pressure can also influence codon usage (He et al., 2016).

GC12-GC3 is an indicator of the genomic trend of non-neutral mutational pressure in chloroplast codons; some research indicates that the factors influencing chloroplast codon biases are complex (Chen et al., 2004). The neutrality plot analysis showed that the correlation between GC12 and GC3 was weak, and the slopes of all the trend lines were less than 0.2. Therefore, the influence of mutation on the codons was very small, whereas other factors, such as natural selection, may have more effect on codon preference (Galtier et al., 2018). In summary, natural selection and gene base composition are the main factors affecting CUB; similar conclusions were also obtained in a study of mitochondrial codons, a finding which was consistent with the selection–mutation drift theory (Kawabe & Miyashita, 2003).

## Conclusions

The complete chloroplast genomes of *B. papyrifera*, *B. kazinoki*, and *B. kaempferi* were 160,239 bp, 160,841 bp, and 160,592 bp in length, respectively. The *RPL22* gene was incomplete or missing in *Broussonetia*. *M. scandens* was clustered within the genus *Broussonetia*, with very high bootstrap value. The indices of codon usage suggested that natural selection and gene base composition are the main factors affecting codon usage in the cp genomes of members of the family Moraceae, a finding which is consistent with the selection–mutation drift theory.

##  Supplemental Information

10.7717/peerj.14293/supp-1 Supplemental Information 1 The nucleotide diversity (pi) values of the aligned 66 protein-coding genes (PCGs) within 35 Moraceae cp genomes The genes from the inverted repeat regions (IR) are indicated by red, genes left and right of which are from large single copy regions (LSC) and small single copy regions (SSC), respectively.

10.7717/peerj.14293/supp-2 Supplemental Information 2 Supplemental Tables

10.7717/peerj.14293/supp-3 Supplemental Information 3 The code used to get CDS

10.7717/peerj.14293/supp-4 Supplemental Information 4 The tre format file of Fig. 4 (Phylogenetic trees from maximum likelihood (ML) analyses of 35 species based on 66 protein-coding genes) is generated by RAxML This file can be read by TreeGraph to draw phylogenetic trees.

10.7717/peerj.14293/supp-5 Supplemental Information 5 The tre format file of Fig. 5 (Phylogenomic analysis of the divergence time and natural selection) is generated by PAML This file can be read by FigTree to draw phylogenetic trees with divergence time.

## Additional Information and Declarations

### DNA Deposition

The following information was supplied regarding the deposition of DNA sequences: The complete cp genomes sequences of *B. papyrifera*, *Broussonetia kazinoki*, and *Broussonetia kaempferi* are available at GenBank: NC_035569.1, MH223642.1, MH223641.1.

### Data Availability

The following information was supplied regarding data availability: The data is available at NCBI SRA: PRJNA817344.

## References

[ref-1] Ahmad T, Sablok G, Tatarinova TV, Xu Q, Deng XX, Guo WW (2013). Evaluation of codon biology in Citrus and *Poncirus trifoliata* based on genomic features and frame corrected expressed sequence tags. DNA Research.

[ref-2] Asaf S, Khan AL, Khan MA, Shahzad R, Lubna, Kang SM, Al-Harrasi A, Al-Rawahi A, Lee IJ (2018). Complete chloroplast genome sequence and comparative analysis of loblolly pine (*Pinus taeda* L.) with related species. PLOS ONE.

[ref-3] Athey J, Alexaki A, Osipova E, Rostovtsev A, Santana-Quintero LV, Katneni U, Simonyan V, Kimchi-Sarfaty C (2017). A new and updated resource for codon usage tables. BMC Bioinformatics.

[ref-4] Bakker FT, Le D, Yu J, Mohammadin S, Wei Z, van de Kerke S, Gravendeel B, Nieuwenhuis M, Staats M, Alquezar-Planas DE, Holmer R (2016). Herbarium genomics: plastome sequence assembly from a range of herbarium specimens using an Iterative Organelle Genome Assembly pipeline. Biological Journal of the Linnean Society.

[ref-5] Bendich AJ (1987). Why do chloroplasts and mitochondria contain so many copies of their genome. Bioessays.

[ref-6] Cao XX, Yang LG, Xue Q, Yao F, Sun J, Yang FY, Liu YJ (2020). Antioxidant evaluation-guided chemical profiling and structure–activity analysis of leaf extracts from five trees in *Broussonetia* and *Morus* (Moraceae). Scientific Reports.

[ref-7] Chang CS, Liu HL, Moncada X, Seelenfreundd A, Seelenfreunde D, Chung KF (2015). A holistic picture of Austronesian migrations revealed by phylogeography of Pacific paper mulberry. Proceedings of the National Academy of Sciences of the Unite States of America.

[ref-8] Chen SL, Lee W, Hottes AK, Shapiro L, McAdams HH (2004). Codon usage between genomes is constrained by genome-wide mutational processes. Proceedings of the National Academy of Sciences of the Unite States of America.

[ref-9] Chen Y, Ye W, Zhang Y, Xu Y (2015). High speed BLASTN: an accelerated Mega BLAST search tool. Nucleic Acids Research.

[ref-10] Chung KF, Kuo WH, Hsu YH, Li YH, Rubite RR, Xu WB (2017). Molecular recircumscription of *Broussonetia* (Moraceae) and the identity and taxonomic status of *B. kaempferi* var australis. Botanical Studies.

[ref-11] Comeron JM, Aguadé M (1998). An evaluation of measures of synonymous codon usage bias. Journal of Molecular Evolution.

[ref-12] Cove D (2000). The moss Physcomitrella patens. Journal of Plant Growth Regulation.

[ref-13] Darriba D, Taboada GL, Doallo R, Posada D (2012). JModelTest 2: more models, new heuristics and parallel computing. Nature Methods.

[ref-14] Fernández-Mazuecos M, Vargas P (2010). Ecological rather than geographical isolation dominates quaternary formation of Mediterranean *Cistus* species. Molecular Ecology.

[ref-15] Galtier N, Roux C, Rousselle M, Romiguier J, Figuet E, Glémin S, Bierne N, Duret L (2018). Codon usage bias in animals: disentangling the effects of natural selection effective population size and GC-biased gene conversion. Molecular Biology and Evolution.

[ref-16] Gantt JS, Baldauf SL, Calie PJ, Weeden NF, Palmer JD (1991). Transfer of *rpl22* to the nucleus greatly preceded its loss from the chloroplast and involved the gain of an intron. The EMBO Journal.

[ref-17] Gao X, Zhang X, Meng H, Li J, Zhang D, Liu C (2018). Comparative chloroplast genomes of *Paris* Sect. Marmorata: insights into repeat regions and evolutionary implications. BMC Genomics.

[ref-18] Gertz EM, Yu YK, Agarwala R, Schäffer AA, Altschul SF (2006). Composition-based statistics and translated nucleotide searches: improving the TBLASTN module of BLAST. BMC Biology.

[ref-19] Green PS (1993). Notes relating to the floras of Norfolk & Lord Howe Islands IV. Kew Bulletin.

[ref-20] Guisinger MM, Chumley TW, Kuehl JV, Boore JL, Jansen RK (2010). Implications of the plastid genome sequence of typha (*Typhaceae poales*) for understanding genome evolution in Poaceae. Journal of Molecular Evolution.

[ref-21] Hazzi NA, Moreno JS, Ortiz-Movliav C, Palacio RD (2018). Biogeographic regions and events of isolation and diversification of the endemic biota of the tropical andes. Proceedings of the National Academy of Sciences of the Unite States of America.

[ref-22] He B, Dong H, Jiang C, Cao F, Tao S, Xu LA (2016). Analysis of codon usage patterns in *Ginkgo biloba* reveals codon usage tendency from A/U-ending to G/C-ending. Scientific Reports.

[ref-23] Hershberg R, Petrov DA (2008). Selection on codon bias. Annual Review of Genetics.

[ref-24] Huang CY, Ayliffe MA, Timmis JN (2003). Direct measurement of the transfer rate of chloroplast DNA into the nucleus. Nature.

[ref-25] James BR (2003). Ancient horizontal gene transfer. Nature Reviews Genetics.

[ref-26] Jansen RK, Cai Z, Raubeson LA, Daniell H, Depamphilis CW, Leebens-Mack J, Müller KF, Guisinger-Bellian M, Haberle RC, Hansen AK, Chumley TW, Lee SB, Peery R, McNeal JR, Kuehl JV, Boore JL (2007). Analysis of 81 genes from 64 plastid genomes resolves relationships in angiosperms and identifies genome-scale evolutionary patterns. Proceedings of the National Academy of Sciences of the Unite States of America.

[ref-27] Jiao F, Luo R, Dai X, Liu H, Yu G, Han S, Lu X, Su C, Chen Q, Song Q, Meng C, Li F, Sun H, Zhang R, Hui T, Qian Y, Zhao A, Jiang Y (2020). Chromosome-level reference genome and population genomic analysis provide insights into the evolution and improvement of domesticated mulberry (*Morus alba*). Molecular Plant.

[ref-28] Jin JJ, Yu WB, Yang JB, Song Y, De Pamphilis CW, Yi TS, Li DZ (2020). GetOrganelle: a fast and versatile toolkit for accurate de novo assembly of organelle genomes. Genome Biology.

[ref-29] Kawabe A, Miyashita NT (2003). Patterns of codon usage bias in three dicot and four monocot plant species Genes. Genes & Genetic Systems.

[ref-30] Kent WJ (2002). BLAT—the BLAST-like alignment tool. Genome Research.

[ref-31] Kong W, Yang J (2016). The complete chloroplast genome sequence of *Morus mongolica* and a comparative analysis within the Fabidae clade. Current Genetics.

[ref-32] Kuo WH, Liu SH, Chang CC, Hsieh CL, Li YH, Ito T, Won H, Kokubugata G, Chung KF (2022). Plastome phylogenomics of *Allaeanthus*, Broussonetia and *Malaisia* (Dorstenieae, Moraceae) and the origin of *B.*×* kazinoki*. Journal of Plant Research.

[ref-33] Langmead B, Salzberg SL (2012). Fast gapped-read alignment with Bowtie 2. Nature Methods.

[ref-34] Li H, Handsaker B, Wysoker A, Fennell T, Ruan J, Homer N, Marth G, Abecasis G, Durbin R (2009). The sequence alignment/map (SAM) format and SAMtools. Bioinformatics.

[ref-35] Li H, Ruan J, Durbin R (2008). Mapping short DNA sequencing reads and calling variants using mapping quality scores. Genome Research.

[ref-36] Li X, Zhao Y, Tu X, Li C, Zhu Y, Zhong H, Liu ZJ, Wu S, Zhai J (2021). Comparative analysis of plastomes in Oxalidaceae: phylogenetic relationships and potential molecular markers. Plant Diversity.

[ref-37] Li ZH, Jiang Y, Ma X, Li JW, Yang JB, Wu JY, Jin XH (2020). Plastid genome evolution in the subtribe calypsoinae (*Epidendroideae Orchidaceae*). Genome Biology and Evolution.

[ref-38] Liu X (2013). A more accurate relationship between ‘effective number of codons’ and GC3s under assumptions of no selection. Computational Biology and Chemistry.

[ref-39] Luo R, Liu B, Xie Y, Li Z, Huang W, Yuan J, He G, Chen Y, Pan Q, Liu Y, Tang J, Wu G, Zhang H, Shi Y, Liu Y, Yu C, Wang B, Lu Y, Han C, Cheung DW, Yiu SM, Peng S, Zhu X, Liu G, Liao X, Li Y, Yang H, Wang J, Lam TW, Wang J (2012). SOAPdenovo2: an empirically improved memory-efficient short-read de novo assembler. GigaScience.

[ref-40] Mao Q, Bi G (2016). Complete chloroplast genome of *Ficus racemosa* (Moraceae). Mitochondrial DNA. Part A, DNA Mapping, Sequencing and Analysis.

[ref-41] Matisoo-Smith EA (2015). Tracking Austronesian expansion into the Pacific via the paper mulberry plant. Proceedings of the National Academy of Sciences of the Unite States of America.

[ref-42] Millen RS, Olmstead RG, Adams KL, Palmer JD, Lao NT, Heggie L, Kavanagh TA, Hibberd JM, Gray JC, Morden CW, Calie PJ, Jermiin LS, Wolfe KH (2001). Many parallel losses of *infA* from chloroplast DNA during angiosperm evolution with multiple independent transfers to the nucleus. The Plant Cell.

[ref-43] Mugal CF, Wolf JB, Kai I (2014). Why time matters: codon evolution and the temporal dynamics of dN/dS. Molecular Biology and Evolution.

[ref-44] Muse SV (2000). Examining rates and patterns of nucleotide substitution in plants. Plant Molecular Biology.

[ref-45] Nie X, Deng P, Feng K, Liu P, Du X, You FM, Song W (2014). Comparative analysis of codon usage patterns in chloroplast genomes of the Asteraceae family. Plant Molecular Biology Reporter.

[ref-46] Payacan C, Moncada X, Rojas G, Clarke A, Chung KF, Allaby R, Seelenfreund D, Seelenfreund D (2017). Phylogeography of herbarium specimens of asexually propagated paper mulberry [*Broussonetia papyrifera* (L) L’Hér ex Vent (Moraceae)] reveals genetic diversity across the Pacific. Annals of Botany.

[ref-47] Peden JF (1999). Analysis of codon usage. PhD Dissertation.

[ref-48] Peng F, Zhao Z, Xu B, Han J, Yang Q, Lei Y, Tian B, Liu Z (2020). Characteristics of organellar genomes and nuclear internal transcribed spacers in the tertiary relict genus *Dipelta* and their phylogenomic implications. Frontiers in Genetics.

[ref-49] Peng X, Liu H, Chen P, Tang F, Hu Y, Wang F, Zhao MPiZ, Chen N, Chen H, Zhang X, Yan X, Liu M, Fu X, Zhao G, Yao P, Wang L, Dai H, Li X, Xiong W, Xu W, Zheng H, Yu H, Shen SAA (2019). Chromosome-scale genome assembly of paper mulberry (*Broussonetia papyrifera*) provides new insights into its forage and papermaking usage. Molecular Plant.

[ref-50] Pond SL, Frost SD, Muse SV (2005). HyPhy: hypothesis testing using phylogenies. Bioinformatics.

[ref-51] Qin Z, Cai Z, Xia G, Wang M (2013). Synonymous codon usage bias is correlative to intron number and shows disequilibrium among exons in plants. BMC Genomics.

[ref-52] Rambaut A, Drummond AJ, Xie D, Baele G, Suchard MA (2018). Posterior summarization in Bayesian phylogenetics using Tracer 1.7. Systematic Biology.

[ref-53] Ranwez V, Douzery EJP, Cambon C, Chantret N, Delsuc F (2018). MACSE v2: toolkit for the alignment of coding sequences accounting for frameshifts and stop codons. Molecular Biology and Evolution.

[ref-54] Ronquist F, Teslenko M, van der Mark P, Ayres DL, Darling A, Höhna S, Larget B, Liu L, Suchard MA, Huelsenbeck JP (2012). MrBayes 3.2: efficient Bayesian phylogenetic inference and model choice across a large model space. Systematic Biology.

[ref-55] Rozas J, Ferrer-Mata A, Sánchez-DelBarrio JC, Guirao-Rico S, Librado P, Ramos-Onsins SE, Sánchez-Gracia A (2017). DnaSP 6: DNA sequence polymorphism analysis of large datasets. Molecular Biology and Evolution.

[ref-56] Schattner P, Brooks AN, Lowe TM (2005). The tRNAscan-SE snoscan and snoGPS web servers for the detection of tRNAs and snoRNAs. Nucleic Acids Research.

[ref-57] Seelenfreund D, Piña R, Ho KY, Lobos S, Moncada X, Seelenfreund A (2011). Molecular analysis of *Broussonetia papyrifera* (l) vent (*magnoliophyta: urticales*) from the pacific based on ribosomal sequences of nuclear DNA. New Zealand Journal of Botany.

[ref-58] Sharp PM, Li WH (1986). An evolutionary perspective on synonymous codon usage in unicellular organisms. Journal of Molecular Evolution.

[ref-59] Shi C, Hu N, Huang H, Gao J, Zhao YJ, Gao LZ (2012). An improved chloroplast DNA extraction procedure for whole plastid genome sequencing. PLOS ONE.

[ref-60] Si B, Xu W, Zhang X, Guo J, Diao Q, Tu Y (2018). Influence of different additives on fermentation quality of hybrid paper mulberry silage. Chinese Journal of Animal Nutrition.

[ref-61] Song WC, Ji CX, Chen ZM, Cai HH, Wu XM, Shi C, Wang S (2022). Comparative analysis the complete chloroplast genomes of nine *Musa* Species: genomic features, comparative analysis, and phylogenetic implications. Frontiers in Plant Science.

[ref-62] Stamatakis A (2014). RAxML version 8: a tool for phylogenetic analysis and post-analysis of large phylogenies. Bioinformatics.

[ref-63] Stegemann S, Bock R (2006). Experimental reconstruction of functional gene transfer from the tobacco plastid genome to the nucleus. The Plant Cell.

[ref-64] Tamura K, Stecher G, Kumar S (2021). MEGA11: molecular evolutionary genetics analysis version 11. Molecular Biology and Evolution.

[ref-65] Tao X, Ma L, Zhang Z, Liu W, Liu Z (2017). Characterization of the complete chloroplast genome of alfalfa (*Medicago sativa*) (Leguminosae). Gene Reports.

[ref-66] Thompson JD, Gibson TJ, Plewniak F, Jeanmougin F, Higgins DG (1997). The CLUSTAL_X windows interface: flexible strategies for multiple sequence alignment aided by quality analysis tools. Nucleic Acids Research.

[ref-67] Ueda M, Nishikawa T, Fujimoto M, Takanashi H, Arimura SI, Tsutsumi N, Kadowaki KI (2008). Substitution of the gene for chloroplast *RPS16* was assisted by generation of a dual targeting signal. Molecular Biology and Evolution.

[ref-68] Wang GW, Huang BK, Qin LP (2012). The genus *Broussonetia*: a review of its phytochemistry and pharmacology. Phytotherapy Research.

[ref-69] Wang YH, Wicke S, Wang H, Jin JJ, Chen SY, Zhang SD, Li DZ, Yi TS (2018). Plastid genome evolution in the early-diverging legume subfamily Cercideae (Fabaceae). Frontiers in Plant Science.

[ref-70] Wright F (1990). The ‘effective number of codons’ used in a gene. Gene.

[ref-71] Wu Y, Liu F, Yang DG, Li W, Zhou XJ, Pei XY, Liu YG, He KL, Zhang WS, Ren ZY, Zhou KH, Ma XF, Li ZH (2018). Comparative chloroplast genomics of *Gossypium* species: insights into repeat sequence variations and phylogeny. Frontiers in Plant Science.

[ref-72] Wyman SK, Jansen RK, Boore JL (2004). Automatic annotation of organellar genomes with DOGMA. Bioinformatics.

[ref-73] Xu Q, Xiong G, Li P, He F, Huang Y, Wang K, Li Z, Hua J (2012). Analysis of complete nucleotide sequences of 12 *Gossypium* chloroplast genomes: origin and evolution of allotetraploids. PLOS ONE.

[ref-74] Yang Z (2007). PAML 4: phylogenetic analysis by maximum likelihood. Molecular Biology and Evolution.

[ref-75] Yu H, Han A, Wang C, Li Y, Liu L (1997). Nuclear genomic homologous sequence of chloroplast *psba* gene in rice (*oryza sativa*, L). Journal of Tropical and Subtropical Botany.

[ref-76] Zhang T, Qi G, Ye H, Zhang M, Xiao W, Yuan Z (2017). Codon usage bias in pomegranate transcriptome. Acta Horticulturae Sinica.

[ref-77] Zhang W, Yang G, Zhao Y, Xu Z, Huang H, Zhou J (2022). The chloroplast genome comparative characteristic of artificial breeding tree, a case about *Broussonetia Kazinoki* x *Broussonetia Papyifera*. BIOCELL.

[ref-78] Zhang XS, Cao ZY, Wu ZY, Chen XQ (1998). MORACEAE. Flora of China.

[ref-79] Zheng H, Huang B, Qin L, Zhang Q (2002). Biological character and resources distribution of Broussonetia. Chinese Wild Plant Resources.

[ref-80] Zhou M, Long W, Li X (2008). Patterns of synonymous codon usage bias in chloroplast genomes of seed plants. Forest Study of China.

