# Supplementary figures and images for: The complete chloroplast genome sequences of three Broussonetia species and comparative analysis within the Moraceae
Source: PeerJ. 2022 Oct 31;10:e14293. doi: 10.7717/peerj.14293 (PMC9632464; doi:10.7717/peerj.14293)

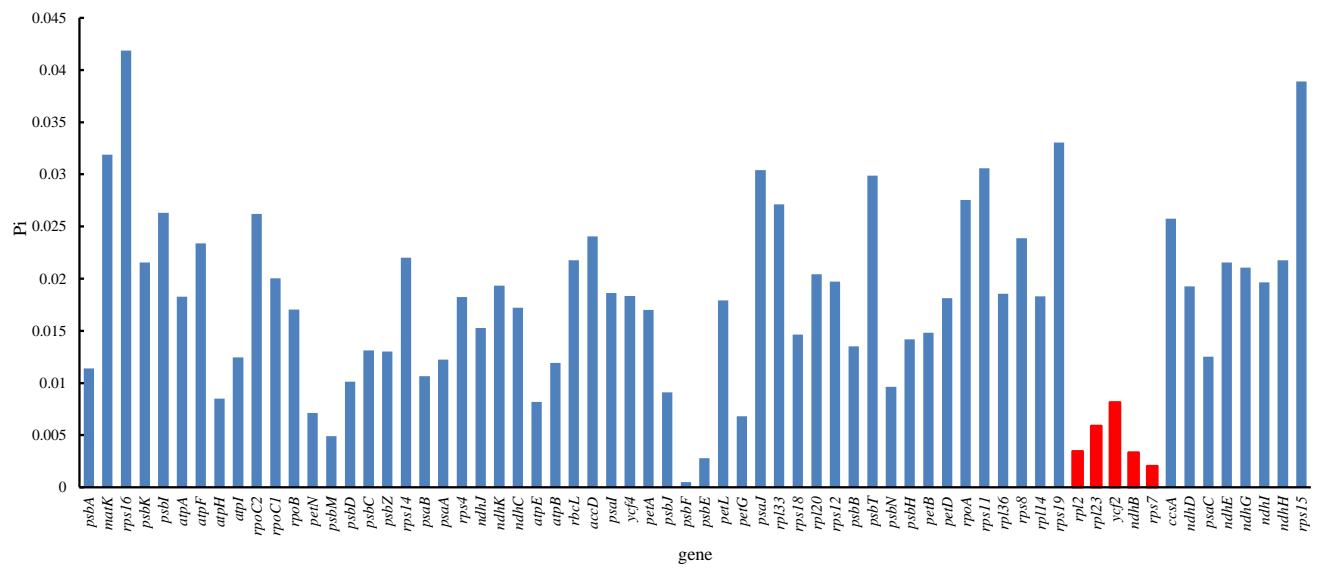

Supplement: Supplemental Information 1 — The genes from the inverted repeat regions (IR) are indicated by red, genes left and right of which are from large single copy regions (LSC) and small single copy regions (SSC), respectively. [file peerj-10-14293-s001.pdf]
